# A Nanosensor for TNT Detection Based on Molecularly Imprinted Polymers and Surface Enhanced Raman Scattering

**DOI:** 10.3390/s110302700

**Published:** 2011-03-01

**Authors:** Ellen L. Holthoff, Dimitra N. Stratis-Cullum, Mikella E. Hankus

**Affiliations:** United States Army Research Laboratory, RDRL-SEE-O, 2800 Powder Mill Road, Adelphi, MD 20783, USA; E-Mails: dimitra.stratiscullum1@us.army.mil (D.N.S.-C.); mikella.hankus@us.army.mil (M.E.H.)

**Keywords:** molecular imprinting, surface enhanced Raman scattering, sensor, explosives detection

## Abstract

We report on a new sensor strategy that integrates molecularly imprinted polymers (MIPs) with surface enhanced Raman scattering (SERS). The sensor was developed to detect the explosive, 2,4,6-trinitrotoluene (TNT). Micron thick films of sol gel-derived xerogels were deposited on a SERS-active surface as the sensing layer. Xerogels were molecularly imprinted for TNT using non-covalent interactions with the polymer matrix. Binding of the TNT within the polymer matrix results in unique SERS bands, which allow for detection and identification of the molecule in the MIP. This MIP-SERS sensor exhibits an apparent dissociation constant of (2.3 ± 0.3) × 10^−5^ M for TNT and a 3 μM detection limit. The response to TNT is reversible and the sensor is stable for at least 6 months. Key challenges, including developing a MIP formulation that is stable and integrated with the SERS substrate, and ensuring the MIP does not mask the spectral features of the target analyte through SERS polymer background, were successfully met. The results also suggest the MIP-SERS protocol can be extended to other target analytes of interest.

## 1. Introduction

Rapid detection and identification of energetic materials is a priority for military and homeland defense applications with the increased need to avoid potential harm caused by explosive hazards. An analyte of particular interest is 2,4,6-trinitrotolune (TNT), a commonly used explosive in the preparation of landmines for military and terrorist activities. Several methodologies for TNT detection have been reported, including fluorescent polymers [1–3], microcantilevers [4–6], ion mobility spectrometry [7–9], and Raman spectroscopy [10–12]. Although successfully employed, current capabilities have limitations. For example, these technologies may not always be specific or extendable to other chemical systems, and may only allow for bulk material assessment.

In the current work, we report on a spectroscopic method that aims to address many of the above limitations using molecularly imprinted sol-gel derived xerogels in combination with surface enhanced Raman scattering (SERS). Molecular imprinting involves arranging polymerizable functional monomers around a template, followed by polymerization and template removal [13]. Molecularly imprinted polymers (MIPs) can be utilized as artificial recognition elements for target chemical analytes of interest and there are numerous reports detailing the use of this technique for the preparation of polymers which have the ability to bind a specific chemical target [14–18]. Here, molecular imprinting of TNT in xerogels was achieved using a non-covalent imprinting approach [19]. Polymerizable monomers (*i.e.*, precursors) were chosen based on potential non-covalent interactions with the TNT molecules, allowing for increased target recognition [20]. The xerogel matrix included 3-aminopropyltriethoxysilane (APTES), which has been shown to engage in strong non-covalent interactions with TNT molecules [21] via the formation of a charge-transfer complex between the electron-deficient aromatic ring of the nitro-aromatic species and the electron-rich amino group of the precursor [3,22]. This interaction significantly improves polymer selectivity and affinity for TNT [23].

**Figure 1.**
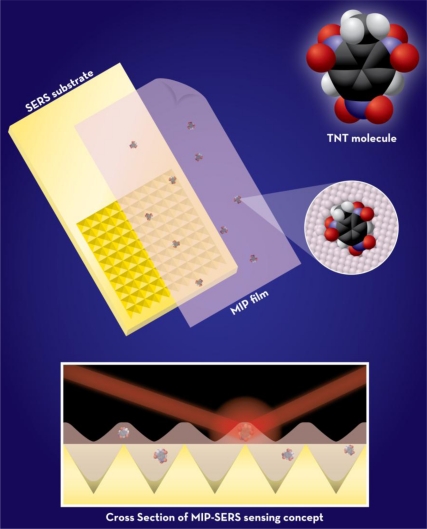
MIP-SERS detection concept.

In chemical sensing applications, a MIP alone does not meet the requirements for a sensor without some form of a transducer to convert the analyte interaction into a measureable signal. There is increasing evidence in the literature of a variety of electrochemical and optical transduction techniques applied to convert a MIP into a sensor for TNT detection [23–27]. In this work, SERS [28] was used as the transduction method to achieve a high level of selectivity. This technique provides vibrational spectra with unique chemical and structural information for a given species. SERS is an extremely sensitive and selective technique that involves enhancements in the Raman scattering intensities of analytes adsorbed on a roughened metal surface (typically, gold or silver) [29,30]. These enhancements (up to 14 orders of magnitude as compared to spontaneous Raman) are due to chemical and electromagnetic enhancement, which results when the incident light in the experiment strikes the metal surface and excites localized surface plasmons. The detection capabilities of SERS make it an excellent transduction method for selective, full compound identification, a capability that is not currently possible with existing MIP sensors. Compared to other, more conventional spectroscopic techniques employed with MIPs, SERS should be less affected by cross-selectivity resulting from non-specific adsorption to the polymer. The basic MIP-SERS detection concept is illustrated in Figure 1. It is important to note that SERS-based techniques alone have not typically proven to be useful for explosives detection. The primary concern is that any chemical components that enter the enhancing field can potentially contribute to the resulting spectra, making real-world samples difficult to differentiate, even with advanced chemometric analysis tools. However, by employing the developed approach presented here, the MIP will concentrate the target to the SERS-active surface, thereby making the combined approach more highly selective than a SERS-only detection platform and free from errors related to background interference.

This integrated MIPs and SERS concept is a novel approach to chemical sensing; however reported investigations of this pairing are scarce [31,32]. The Kantarovich group used a nano fountain pen to print MIP droplets on SERS-active surfaces and directly monitor the uptake and release of the β-blocking drug propranolol by SERS [31]. Kostrewa *et al.* prepared MIPs on SERS-active surfaces to directly monitor the uptake and release of either (2S,3S)-(+)-di-O-benzoyl-tartaric acid or N-benzyloxycarbonyl-(L)-aspartic acid to the polymer by SERS [32]. In this instance, adhesion of the MIP to the SERS-active substrates was unsatisfactory for practical application as the MIP was not truly integrated with the substrate. Herein, we consider adhesion of the polymer to the SERS substrate. Xerogel precursors were chosen based on potential interactions with the metallic under layer of the substrate. The xerogel matrix included 3-mercaptopropyltrimethoxysilane (MPTMS), which contains a thiol group and results in chemisorption of the polymer to the metal surface of the SERS substrate. Films of molecularly imprinted xerogels were deposited on SERS substrates to create a sensor for TNT. Sensitivity is determined by the SERS substrate used in this study, while selectivity is provided by both the specific binding interaction of the TNT with the MIP and the unique molecular “fingerprint” provided by the SERS measurement.

## 2. Experimental Section

### 2.1. Reagents and Materials

APTES, methyltriethoxysilane (C1-TriEOS), and MPTMS were obtained from Gelest. TNT, 2,4-dinitrotoluene (2,4-DNT), 2,6-dinitrotoluene (2,6-DNT), and 1,3-dinitrobenzene (1,3-DNB) were purchased from Cerilliant. Ethanol, acetonitrile, acetic acid, and HCl were obtained from Sigma-Aldrich.

Klarite^®^ substrates were purchased from Renishaw Diagnostics. These substrates consist of a smooth border and a SERS-active patterned grid area. Both surfaces are gold-coated. To protect against environmental and shipping hazards, each substrate is placed in a separate microscope slide holder enclosed within an opaque vacuum-sealed pouch before shipping. Just prior to use, each substrate was removed from the vacuum-sealed pouch and slide holder. The substrates were used as received.

All solvents were HPLC grade. All chemicals were used as received unless otherwise noted.

### 2.2. Instrumentation

SERS data was recorded using a Renishaw inVia Reflex Raman microscope equipped with a near-infrared diode laser excitation source (λ = 785 nm). The light from the diode was focused onto the samples at the microscope stage through a 20× objective. Prior to coupling into the microscope, the diode laser beam was circularized by inserting a pinhole into the optical beam path and neutral density filters were used resulting in reduction of the maximum available laser power to 17 mW. Samples at the microscope stage were positioned remotely with a joystick using an encoded, motorized *XYZ* translation stage (0.1 μm step size) controlled by a Prior Scientific ProScan II controller. WiRE 2.0 software, operating on a bench top computer, was used for instrument control and data collection. Before all measurements, the instrument was wavelength calibrated using an internal silicon standard.

Films were produced by spin casting with a spin coater (Laurell Technologies, model WS-400B-6NPP/LITE).

### 2.3. SERS Measurements

A stock solution of TNT was prepared at 4.0 × 10^−4^ M, in acetonitrile. All xerogel films were incubated in this solution at room temperature for 24 h. The xerogels were subsequently rinsed with acetonitrile (200 μL) to remove residual TNT from the surface. Stock solutions of TNT, 2,4-DNT, 2,6-DNT, and 1,3-DNB were prepared at 7.5 × 10^−5^ M, in acetonitrile, and incubated with the xerogel films in the same manner.

All SERS spectra were acquired using Klarite^®^ substrates. Duplicate samples were prepared for each study and five separate spectral acquisitions were obtained for each sample. Each SERS spectrum was collected over a range from 700 cm^−1^ to 1,500 cm^−1^ with a 10.00 s exposure time and a spectral resolution better than 1 cm^−1^ and is the result of three accumulations. All data are presented as the average (10 measurements) from duplicate samples and corresponding variance represents 1σ.

### 2.4. Overall MIP Fabrication

**Figure 2.**
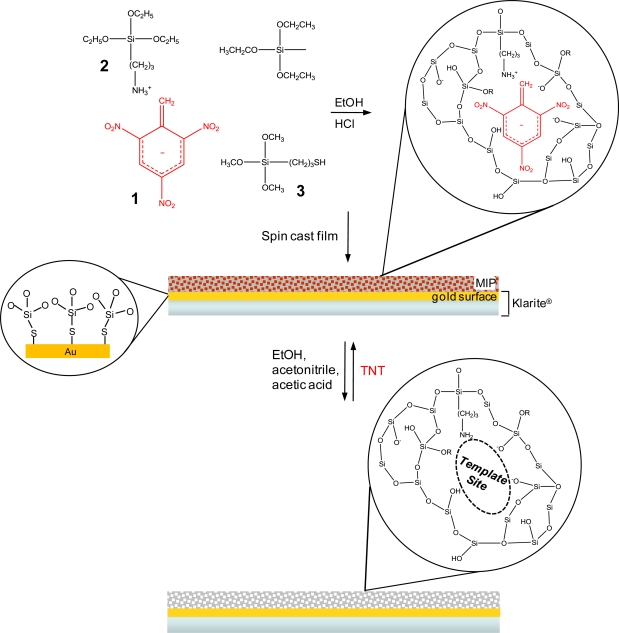
Reaction protocol for producing an integrated MIP-SERS sensor.

The overall MIP production and SERS integration protocol is illustrated in Figure 2 for the template/target molecule, TNT (**1**). Briefly, the TNT-doped sol, comprised of APTES (**2**), MPTMS (**3**), and C1-TriEOS is spun cast onto a Klarite^®^ substrate and allowed to gel, and the xerogel formed. The non-covalent interaction between APTES and TNT is illustrated, as well as chemisorption of the MPTMS to the gold metal layer of the Klarite^®^ substrate. The TNT is removed from the xerogel with a solution containing ethanol, acetonitrile, and acetic acid. The xerogel is then immersed in a target analyte (TNT) solution, filling all the analyte accessible template sites.

### 2.5. TNT-Doped Xerogel Preparation

A TNT stock solution was prepared at 9.85 × 10^−3^ M, in acetonitrile. Sol solutions were prepared by mixing C1-TriEOS (110 μL, 0.552 mmol), MPTMS (2.813 μL, 1.51 × 10^−2^ mmol), APTES (3.50 μL, 1.51 × 10^−2^ mmol), ethanol (1.25 mL, 21.4 mmol), and HCl (6.25 μL of 1 M, 6.25 × 10^−3^ mmol). The C1-TriEOS, MPTMS, APTES, and HCl were added to the ethanol at room temperature and then stirred for 30 min to ensure a visually homogeneous sol solution.

The TNT-doped sol solution was prepared by adding 150 μL of the TNT stock solution to the prehydrolyzed C1-TriEOS/MPTMS/APTES/HCl/ethanol sol solution. This sol solution was then vigorously mixed for 30 s with a touch mixer (Scientific Industries, Vortex-Genie 2).

Xerogel films (7 μm to 10 μm thickness) were formed by spin casting (4,000 rpm, 2 min) a 50 μL aliquot of the final sol mixture onto a Klarite^®^ substrate. The films were aged at room temperature for 2–3 days and were transparent to the eye.

### 2.6. TNT Removal from the Xerogel

TNT was extracted from the xerogel films with an ethanol/acetonitrile/acetic acid (v/v/v 8:2:1) solution. The xerogels were allowed to react with this solution at room temperature for 24 h. All xerogel films were subsequently rinsed with ethanol to remove the residual acidic solvent.

### 2.7. Control Xerogels

A series of control xerogel films were prepared to ensure that the observed sensor response did not arise from artifacts. The controls were prepared by following the exact reaction sequence described above except as noted below. Control A is formed by eliminating TNT. Control B is formed by eliminating TNT and APTES.

## 3. Results and Discussion

There are several key challenges to overcome in the development of a hybrid MIP-SERS sensing platform including: (i) developing a MIP that does not completely mask the spectral features of the target analyte through SERS polymer background; (ii) developing a strategy for MIP integration that enables target interaction within the surface enhanced plasmon field responsible for SERS signal enhancement; (iii) developing a MIP formulation that is stable and truly integrated with the SERS substrate to allow for practical application in the field; and (iv) ensuring that the developed MIP allows for template removal, analyte reintroduction, and also provides selectivity for the target analyte components.

### 3.1. Molecularly Imprinted Xerogels

Different formulations of molecularly imprinted xerogel films were tested where the amino-containing precursor was varied to determine the effect on TNT binding (data not shown). These precursors included 3-(N-allylamino)propyltrimethoxysilane, 4-aminobutyltriethoxysilane, n-butylaminopropyltrimethoxysilane, and APTES. Xerogels containing the APTES precursor exhibited the most effective TNT binding when templated. Therefore the discussion is limited to the results observed for this xerogel formulation.

### 3.2. SERS Integration

**Figure 3.**
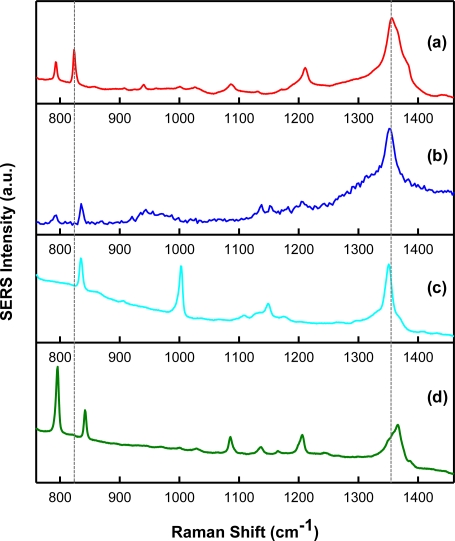
SERS spectral signature of **(a)** TNT, **(b)** 2,4-DNT, **(c)** 1,3-DNB, and **(d)** 2,6-DNT on Klarite^®^. Spectra are offset for clarity. The vertical dashed lines are aligned with the characteristic NO_2_ out-of-plane bending and stretching modes of TNT.

In order to verify molecule templating with SERS, spectra of the chemical analytes and interferents were acquired. Shown in Figure 3(a–d) are intensity-normalized spectra of the analytes investigated during this study. Specifically, the SERS spectra for (a) TNT, (b) 2,4-DNT, (c) 1,3-DNB, and (d) 2,6-DNT (all 1,000 μg/mL, in acetonitrile), over the Raman shift range from 700 cm^−1^ to 1,500 cm^−1^ are presented. As illustrated, this spectral region is interesting since it contains features representative of Raman-active components present in these analytes. In general, the spectra are dominated by SERS-enhanced features centered near 830 cm^−1^ and 1,350 cm^−1^. These bands are a result of NO_2_ out-of-plane bending modes and NO_2_ stretching modes, respectively [33]. These peaks can be used as a “fingerprint” for the detection of these nitro-aromatic compounds by their SERS spectra. Although the key spectral features are comparable, there are minor frequency differences between each nitro-aromatic species. The SERS nitrate stretching modes are observed at 1,356 cm^−1^, 1,350 cm^−1^, 1,366 cm^−1^, and 1,350 cm^−1^ for TNT, 2,4-DNT, 2,6-DNT, and 1,3-DNB, respectively. The absence of the toluene group in 1,3-DNB allows the benzene ring to adsorb more strongly to the substrate surface, leading to a unique feature centered near 1,000 cm^−1^ when compared to the other compounds [34]. These data suggest that it is possible to distinguish these nitro-aromatic species based on their molecular composition, and therefore SERS spectral signatures.

**Figure 4.**
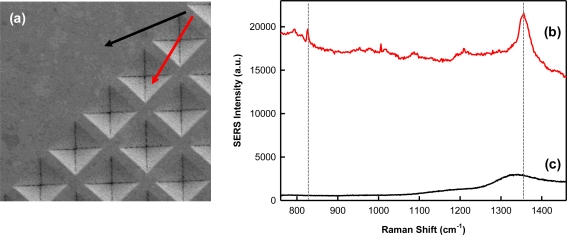
**(a)** SEM image of a Klarite^®^ substrate showing the smooth inactive border and the SERS-active patterned grid area. The black and red arrows illustrate the measurement areas for the Raman and SERS spectra, respectively. SERS **(b)** and Raman **(c)** spectra recorded for a TNT-doped xerogel film. The vertical lines indicate the peaks related to TNT, which are not evident in the Raman spectra.

The integration of MIPs with SERS is not straightforward and poses a unique set of challenges. A major technical challenge is ensuring that the polymer layer is thin and porous enough to concentrate the target analyte within the surface plasmon field responsible for the Raman enhancement, which drops off exponentially with distance from the nanostructured surface. In order to demonstrate SERS enhancement of TNT templated within the polymer matrix, a film was spun cast over both the smooth border (inactive) and SERS-active region of the Klarite^®^ substrate. Figure 4 shows the corresponding Raman and SERS spectra illustrating successful SERS enhancement for TNT (b). The signal from the inactive region (c) does not exhibit TNT and is indicative of Klarite substrate background [30,35]. When incorporated into the polymer, the SERS nitrate stretching mode is shifted to 1,352 cm^−1^. The exact origin of this shift is unclear; however changes in the shifts can be expected if minor structural changes occur when the molecule is encapsulated in a polymer matrix. Kantarovich *et al.* noted a similar observation in the SERS spectra of a MIP that was prepared using (S)-propranolol as a template [31]. Despite these subtle spectral shifts, it is clear from Figure 4 that the polymer background does not prohibit TNT detection. Furthermore, the successful measurement of the TNT template within the MIP shows it is possible to develop a thin and porous MIP film which allows analyte measurement within the critical surface enhancing field distance from the underlying substrate.

**Figure 5.**
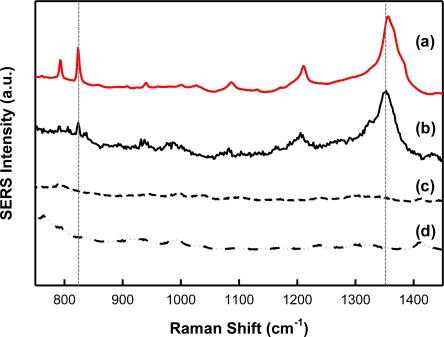
SERS spectra recorded for **(a)** free template TNT, **(b)** TNT-doped xerogel film (MIP), **(c)** control A, and **(d)** control B. Spectra are offset for clarity. The vertical dashed lines indicate the peaks related to TNT, which are not evident in the control spectra.

Figure 5(a–d) presents the SERS spectra for both a TNT-doped xerogel film (b) and control (A and B) xerogel films (c) and (d) spun cast on a Klarite^®^ substrate. Spectrum (b) has been blank corrected by subtracting spectrum (c). A representative SERS spectrum of free TNT on a Klarite^®^ substrate (a) is also provided for reference.

### 3.3. Efficiency of TNT Removal

**Figure 6.**
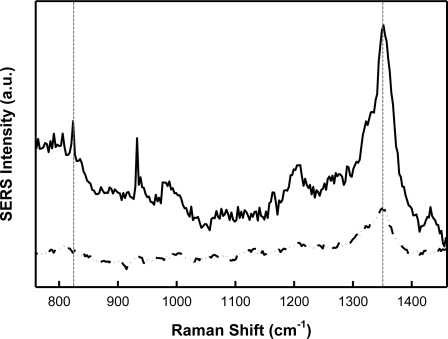
SERS spectra recorded for a TNT-doped xerogel film before (—) and after (– ··–) TNT extraction. Spectra are offset for clarity. The vertical dashed lines indicate the peaks related to TNT, which decreased after template extraction.

Figure 6 presents the SERS spectra for a TNT-doped xerogel film spun cast on a Klarite^®^ substrate measured before (—) and after (– ··–) treatment with the ethanol/acetonitrile/acetic acid extraction solution. Both spectra have been blank corrected by subtracting control A (Figure 5(c)). In the spectrum measured following treatment with the extraction solution, the nitrate stretching mode (1,352 cm^−1^) was reduced extensively and the nitrate bending mode (∼830 cm^−1^) disappeared.

### 3.4. Sensor Response to TNT

**Figure 7.**
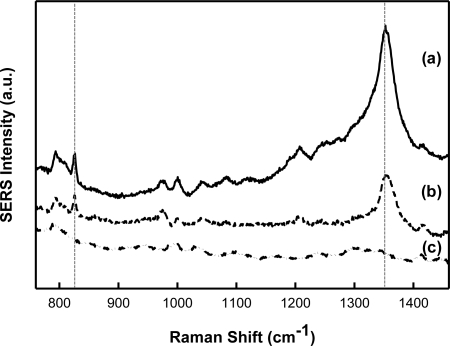
SERS spectra recorded for **(a)** MIP, **(b)** control A, and **(c)** control B after incubation in a 4.0 × 10^−4^ M solution of TNT. Spectra are offset for clarity. The vertical dashed lines indicate the peaks related to TNT, which increased after incubation.

Sensors based on TNT-imprinted xerogel films integrated with Klarite^®^ substrates were tested by exposure to TNT. Specifically, SERS data for both MIP and control (A and B) xerogel films was recorded after incubation in a solution of TNT (4.0 × 10^−4^ M, in acetonitrile). The response is completely reversible after more than 10 cycles with no evidence of signal intensity degradation. These results are summarized in Figure 7(a–c). The SERS TNT fingerprint is apparent in the spectra recorded for the MIP (a) and control A (b). There is no visible TNT fingerprint present in the spectra collected for control B (c). Spectra (a) and (b) have been blank corrected by subtracting control A (Figure 5(c)). Spectrum (c) has been blank corrected by subtracting control B (Figure 5(d)). Due to the presence of APTES in control A and the resulting free amine moieties in the polymer matrix and at the surface, it was expected that TNT would react with this polymer; however it is evident from the recorded SERS spectra that there is preferential binding of TNT to the MIP.

**Figure 8.**
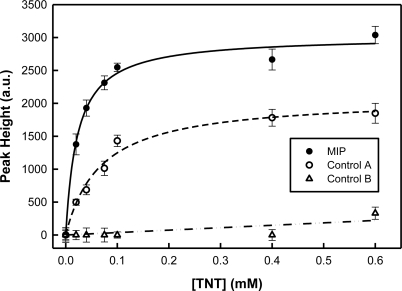
Response profiles for a MIP and control A and B films integrated with a Klarite^®^ substrate.

Figure 8 illustrates the response profile form TNT-responsive and control (A and B) xerogel films integrated with Klarite^®^ substrates to increasing concentrations of TNT. These curves were determined using the height of the spectral band (peak height) resulting from the nitrate stretching mode (1,352 cm^−1^) for both the MIP and controls. Peak heights for the SERS spectral bands associated with the nitrate stretching modes were determined by taking the difference between the peak intensity maximum and an average baseline for each spectrum. This calculation was done after the spectra had been blank corrected. In the MIP, the SERS signal increases as the TNT concentration increases. A single-site saturation ligand binding model (—) yields a dissociation constant of (2.3 ± 0.3) × 10^−5^ M for this molecularly imprinted xerogel for TNT. No significant response is seen from control B when challenged by TNT; however non-specific binding is evident in the results from control A (– –). In this case, the dissociation constant was determined to be (6.7 ± 1.1) × 10^−5^ M. Based on these results, the binding strength of TNT to the MIP is about three times that of control A.

The 3σ limit of detection was determined to be 3.0 μM for this first-generation MIP-SERS sensor for TNT. The achievable sensitivity is limited by the Klarite^®^ SERS substrate. This commercially available substrate was a convenient choice for these seminal studies as it offers reproducibility; however the sensitivity is lacking [35].

### 3.5. Sensor Stability

Adhesion of the MIP to the SERS substrate was assessed by soaking the xerogel films integrated with Klarite^®^ substrates in various aqueous environments (duration ≥ 1 h), including water, 6 M HCl, 0.1 M pH 7.4 phosphate buffer, toluene, and the ethanol/acetonitrile/acetic acid extraction solution. The polymer showed excellent adhesion and stability, with no apparent degradation in sensing performance, which is necessary for practical field use.

We tested the sensor response over a six-month period. The aforementioned analytical figures of merit are reproducible to within <7% relative standard deviation.

### 3.6. Sensor Selectivity

**Figure 9.**
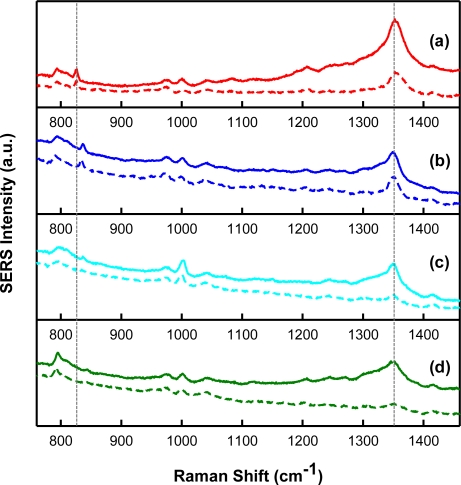
SERS spectra recorded for a MIP after incubation in a 7.5 × 10^−5^ M solution of **(a)** TNT, **(b)** 2,4-DNT, **(c)** 1,3-DNB, and **(d)** 2,6-DNT. The vertical dashed lines are aligned with the characteristic NO_2_ out-of-plane bending and stretching modes of TNT. Spectra are offset for clarity.

To assess the selectivity of the integrated MIP-SERS sensor for the target analyte (*i.e.*, TNT) the sensor was challenged by a series of molecules that are structurally similar to the target molecule. Figure 9(a–d) presents the intensity-normalized SERS spectra for a molecularly imprinted xerogel film (solid lines) and a control A film (dashed lines) integrated with Klarite^®^ substrates recorded after incubation in stock solutions (7.5 × 10^−5^ M, in acetonitrile) of (a) TNT (included for comparison), (b) 2,4-DNT, (c) 1,3-DNB, and (d) 2,6-DNT. All spectra have been blank corrected by subtracting control A (Figure 5(c)). The SERS fingerprints for these compounds are apparent in the spectra recorded for the MIP and are also evident in control A to some extent. There is no visible SERS fingerprint present for any of these analytes in the data collected for control B (data not shown). Due to the presence of APTES in the MIP and control A, it was expected that these nitro-aromatic compounds would react with the polymer; however it is apparent from these spectra that there is preferential binding of TNT to the MIP compared to the other structurally similar molecules. For further comparison, the MIP and control A peak heights of the SERS spectral bands associated with the nitrate stretching modes (∼1,350 cm^−1^) are provided in Table 1. Peak heights were determined by taking the difference between the peak intensity maximum and an average baseline for each spectrum. This calculation was done after the spectra had been blank corrected. The results presented in Figure 9 and Table 1 suggest the MIP has preferential affinity for TNT. The integrated MIP-SERS sensor yields a selectivity factor [18] of 1.63 for TNT over 2,4-DNT, 1.72 for TNT over 1,3-DNB, and 2.12 for TNT over 2,6-DNT.

Additionally, the selectivity can be optimized by considering the unique SERS spectral features associated with these structurally similar compounds. For example, the feature located at 1,000 cm^−1^ is more distinct in Figure 9(c), which suggests the presence of 1,3-DNB. Additionally, comparison of the Raman shifts associated with the NO_2_ out-of-plane bending and stretching modes (∼830 cm^−1^ and ∼1,350 cm^−1^, respectively) for each of these molecules allows for further differentiation. This is evidenced by the differences in the Raman shifts for these NO_2_ modes in Figure 9(a,b). Finally, it is important to note that some selectivity for these structurally similar molecules may be advantageous as 2,4-DNT and 1,3-DNB are manufacturing impurities found in TNT [33] and the detection of these species would be beneficial in recognizing the presence of TNT in the field.

## 4. Conclusions

We have successfully demonstrated a hybrid MIP and SERS sensing concept for the detection of TNT. The first-generation integrated MIP-SERS sensor exhibits a reversible response to the target analyte and is stable in a variety of environments. The results suggest that the MIP-SERS combination is an effective and robust chemical nanosensing scheme. We anticipate the MIP-SERS protocol being extended to include other explosives and chemical warfare agents of interest to the Army. Future work will include incorporation of next generation Klarite^®^ substrates for enhanced sensitivity, additional film optimization for template removal and temporal response studies. A successful MIP-SERS sensing format could reduce sensor cost and size, while maintaining the high sensitivity, selectivity, and portability needed for military applications.

## Figures and Tables

**Table 1.** Peak heights of SERS spectral bands associated with the nitrate stretching modes of various nitro-aromatic compounds.

**Analyte**	**Peak Height**		
	MIP	Control A	Control B
TNT	12,145.05 ± 160.22	5,560.28 ± 226.25	no response
1,4-DNT	7,425.98 ± 274.55	5,581.05 ± 250.54	no response
1,3-DNB	7,038.34 ± 190.38	2,131.46 ± 327.40	no response
2,6-DNT	5,702.42 ± 288.15	1,898.60 ± 267.94	no response
